# Robot‐Assisted Laparoscopic Partial Nephrectomy for Mixed Epithelial and Stromal Tumor of Kidney: A Case Report

**DOI:** 10.1155/criu/5671959

**Published:** 2025-12-20

**Authors:** Sachie Kaichi, Hotaka Matsui, Sayuri Takahashi, Kazuki Akiyama, Daiji Watanabe, Satoru Taguchi, Yuta Yamada, Daisuke Yamada, Mariko Tanaka, Haruki Kume

**Affiliations:** ^1^ Department of Urology, The Institute of Medical Science, The University of Tokyo (IMSUT), Tokyo, Japan; ^2^ Department of Urology, Graduate School of Medicine, The University of Tokyo, Tokyo, Japan, u-tokyo.ac.jp; ^3^ Department of Pathology, Graduate School of Medicine, The University of Tokyo, Tokyo, Japan, u-tokyo.ac.jp

**Keywords:** case report, MEST, partial nephrectomy, robotic surgery

## Abstract

Mixed epithelial and stromal tumor (MEST) of the kidney is a rare and distinctive neoplasm accounting for 0.2% of all renal tumors. The general treatment for MEST typically involves radical nephrectomy or partial nephrectomy. We report a rare case of MEST successfully treated with robot‐assisted partial nephrectomy (RAPN), which, to our knowledge, represents the first documented instance of this approach. The patient is a 53‐year‐old woman with an incidental right renal tumor. The patient visited a primary care physician for chest pain. Computed tomography (CT) scan revealed a 3.5‐cm lesion in the upper pole of the right kidney and was suspected of renal cell carcinoma. The patient was subsequently referred to the department and was admitted to our department for RAPN. We experienced the first case of RAPN performed for MEST of the kidney. Histopathological and immunohistochemical analyses confirmed the diagnosis of MEST. This case highlights the feasibility of robot‐assisted laparoscopic surgery as a minimally invasive treatment option for MEST.

## 1. Introduction

Mixed epithelial and stromal tumor (MEST) of the kidney is a rare and distinctive neoplasm accounting for 0.2% of all renal tumors [1]. MEST of the kidney is a rare tumor first described by Michal and Syrucek in 1998 [2]. Because of its radiological similarity to renal cell carcinoma, MEST is often treated surgically. The general treatment for MEST typically involves radical nephrectomy or partial nephrectomy because it is often diagnosed to be renal cell cancer by radiologists [1]. We present the first case of MEST in which robot‐assisted partial nephrectomy (RAPN) was performed. Here, we describe a case of MEST treated by RAPN and discuss diagnostic considerations and management strategies, including the role of biopsy and observation.

## 2. Case Presentation

The patient is a 53‐year‐old Asian woman. The patient visited a primary care physician for chest pain. A right renal tumor was identified on abdominal contrast‐enhanced computed tomography (CT) scan. The patient was, then, referred to our department. CT scan revealed a 3.5‐cm lesion at the upper pole of the right kidney. The lesion was reported to be a renal cell cancer by radiologists. In July 2023, the patient was admitted to our department for RAPN. Her past medical history includes coronary spasm angina. The patient uses nitroglycerin *prn*. No abnormality was found in the blood tests conducted upon admission. Physical examination was unremarkable. Abdominal ultrasound showed a tumor measuring 3.7 × 2.8 cm in the upper pole of the right kidney. The tumor was mildly hyperechoic on abdominal ultrasound, including cystic areas (Figure 1a). The mass showed mildly high attenuation on noncontrast CT scan. The tumor was composed of cystic and solid components in the CT scan. The solid component showed mildly high absorbance on noncontrast CT scan and was enhanced on contrast CT scan (Figure 1b). The diameter of the tumor seemed to be almost the limit of partial nephrectomy, and there was a high possibility of malignancy by the radiological examination. Therefore, we recommended prompt surgery rather than observation.

Figure 1(a) Abdominal ultrasound showed a tumor measuring 3.7 × 2.8 cm in the upper pole of the right kidney. The tumor was mildly hyperechoic on abdominal ultrasound, including cystic areas. (b) The mass showed mildly high attenuation on noncontrast CT scan. The tumor was composed of cystic and solid components in the CT scan. The solid component showed mildly high absorbance on noncontrast CT scan and was enhanced on contrast CT scan.

**Figure:**
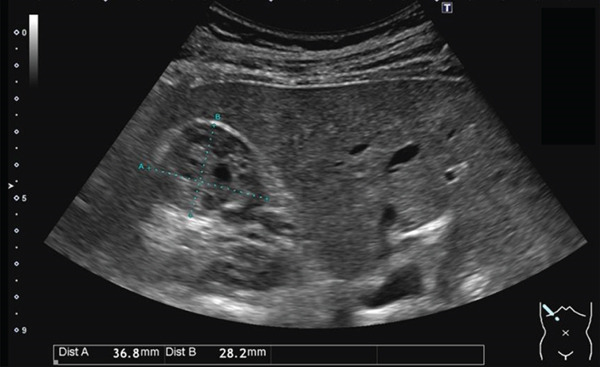
(a)

**Figure:**
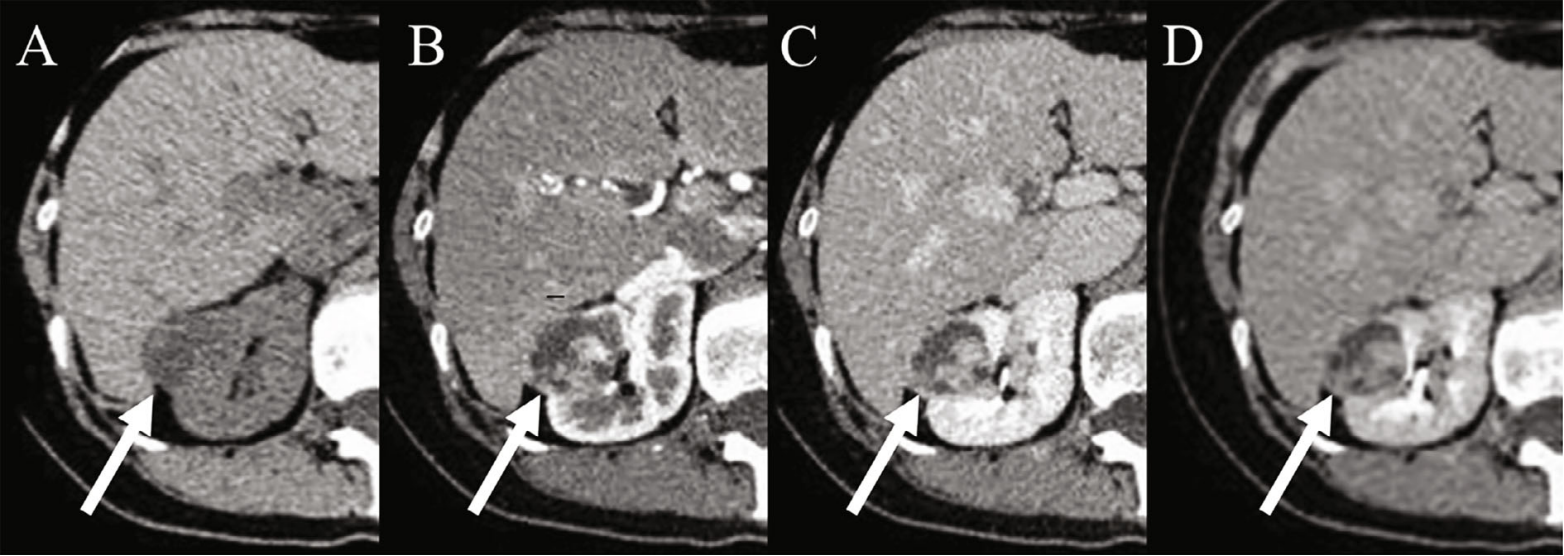
(b)

Hence, the patient underwent RAPN in July 2023.

**Figure 2:**
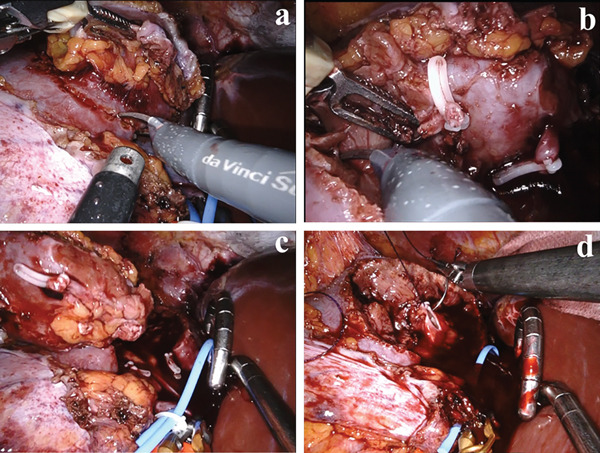
(a–d) The surgery was performed using the Da Vinci Xi Surgical system (Intuitive Surgical, Sunnyvale, CA, United States). The tumor was confirmed by ultrasound and removed along with the surrounding fat tissue.

The surgery was performed with the Da Vinci Xi Surgical system (Intuitive Surgical, Sunnyvale, CA, United States). The tumor was located ventral to the renal vein, and the renal artery and vein were exposed (Figure 2a). The two feeding artery branches that had been identified preoperatively were clipped and cut (Figure 2b). While confirming the tumor using ultrasound, the tumor was marked with perinephric fat, peritoneum, and 3 mm of normal renal tissue. The renal artery was clamped, and the cortex was incised along the markings. The medulla and fat of renal sinus were separated without tumor exposing. The renal calyces were incised and carefully sutured (Figure 2c). Renorrhaphy was also performed after declamping (Figure 2d). A snake retractor was used to avoid liver damage along the incision line of the tumor. The tumor was sent for pathological evaluation. The patient adhered to postoperative follow‐up, and no adverse events were reported.

At the time of this case report, there have been no signs of metastasis or recurrence. The patient expressed satisfaction with the minimally invasive approach and was relieved that kidney function was preserved without recurrence.

Macroscopically, the well‐circumscribed white to yellowish tumor is composed of solid and cystic areas (Figure 3a). Histologically, the tumor was a complex mixture of stromal and epithelial elements. The epithelial components were simple round glands, branching glands, and cysts lined by cuboidal epithelium. The stromal components were composed of slender to plump spindle cells (Figure 3b). Immunohistochemically, the spindle cells were positive for alpha‐smooth muscle actin (*α*SMA), partially positive for estrogen receptor and progesterone receptor, and negative for HMB45 (Figure 3c). The epithelial cells forming glandular structures are positive for cytokeratin 7 and negative for MelanA (Figure 3d). The tumor was diagnosed as a MEST of the kidney.

Figure 3Pathological findings of MESTS. (a) Macroscopically, the tumor was well circumscribed and a mixture of solid and cystic areas. (b) Hematoxylin and eosin staining, ×200. Histologically, the tumor was composed of stromal elements and epithelial elements: The former were spindle cells, and the latter were glands and cysts lined by cuboidal epithelium. (c, d) Immunohistochemically, the spindle cells were positive for alpha‐smooth muscle actin, and the epithelial cells were positive for cytokeratin 7.

**Figure:**
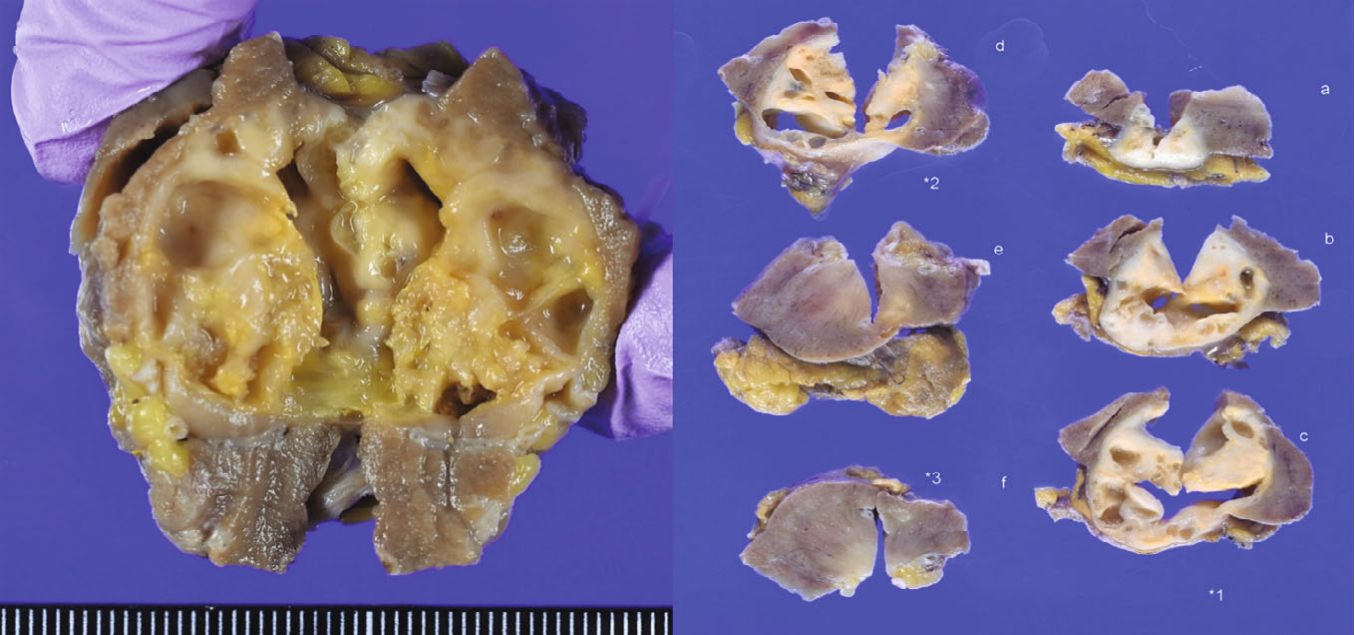
(a)

**Figure:**
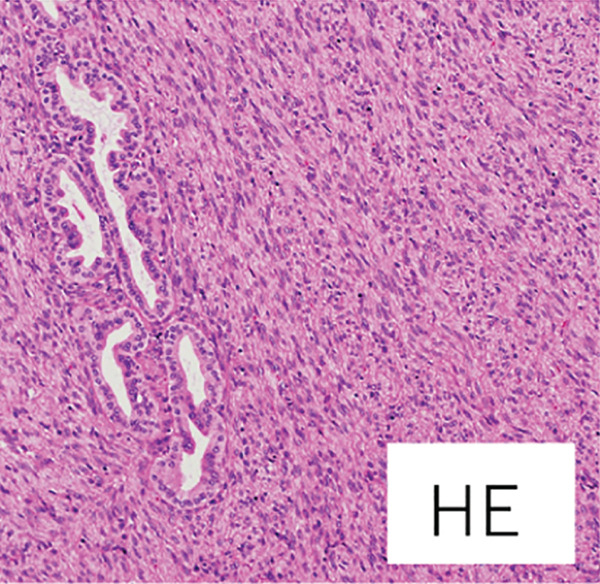
(b)

**Figure:**
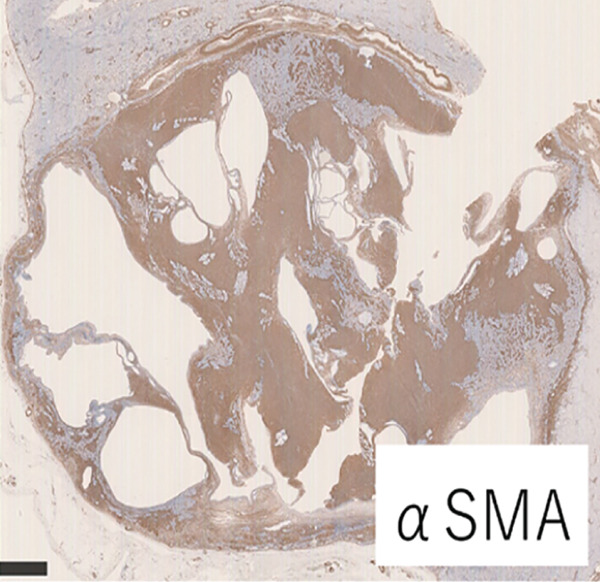
(c)

**Figure:**
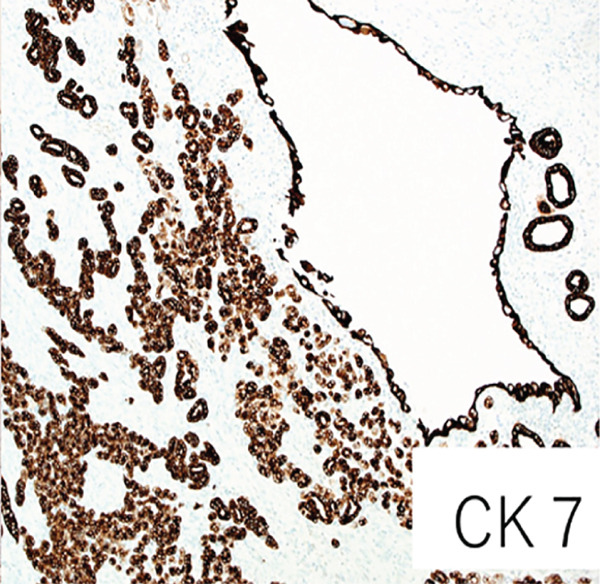
(d)

## 3. Discussion

MEST are characterized by their composition of epithelial components with cystic structures and solid components with spindle‐shaped cells, predominantly found in middle‐aged women. There is a hypothesis suggesting a relationship with estrogen exposure, as evidenced by the expression of estrogen and progesterone receptors in the spindle cells [1]. The spindle cells are often positive for *α*SMA while being negative for angiomyolipoma markers HMB45 and MelanA [3]. Although generally considered to be benign, approximately 15% of cases undergo malignant transformation, requiring surgical resection [4]. Therefore, complete surgical excision remains the standard of care. In our case, preoperative biopsy was considered but avoided because the lesion was radiologically indistinguishable from renal cell carcinoma and the solid portion was small, increasing the risk of sampling error. Previous studies have also suggested that the diagnostic accuracy of renal mass biopsy in cystic lesions is limited [5]. Furthermore, while observation could theoretically be considered for small, incidentally detected renal lesions, the possibility of malignant transformation and patient anxiety favored surgical resection in this case [6].

The characteristic imaging findings of MEST include clear borders, exophytic growth, often presenting as a multilocular cystic tumor with enhancing solid components [7, 8]. It is frequently classified as Bosniak Category III or higher [9]. The stromal component typically demonstrates mild hyperattenuation on plain CT and appears as low signal intensity on T2‐weighted MRI. However, the stromal component often undergoes changes such as edema, fibrosis, or hyalinization. The entire stromal component may not always show low signal intensity on T2‐weighted images [3].

The literature reviews of MEST demonstrate cases of malignant transformation of MEST. While some patients have experienced up to 36 months of recurrence‐free survival [10], others have seen rapid disease progression, with four out of 14 cases resulting in postoperative mortality [11–14]. Two cases were responsive to chemotherapy [4, 10]. In cases undergoing chemotherapy, ifosfamide and doxorubicin were administered for six courses [4]. Reports on malignant MEST cases are rare, and treatment and prognostic factors have yet to be established.

## 4. Conclusion

This is the first case of RAPN performed for a MEST of kidney. Robot‐assisted laparoscopic surgery provides precise dissection and renal preservation, offering a feasible and minimally invasive treatment option. Some MEST cases have reported malignant transformation and postoperative recurrence, emphasizing the need for surgical intervention and strict follow‐up.

**Nomenclature**
MEST: mixed epithelial and stromal tumor
CT: computed tomography
RAPN: robot‐assisted partial nephrectomy

## Ethics Statement

Ethical approval was not required for this case report.

## Consent

Written informed consent was obtained from the patient for publication of this case report.

## Disclosure

All authors reviewed and approved the final manuscript. An ORCID iD for the corresponding author will be provided upon submission, in accordance with the journal′s submission requirements.

## Conflicts of Interest

The authors declare no conflicts of interest.

## Author Contributions

Sachie Kaichi, Sayuri Takahashi, and Hotaka Matsui drafted the manuscript. Daisuke Yamada, Yuta Yamada, and Haruki Kume supervised the surgical management. Kazuki Akiyama, Daiji Watanabe, and Satoru Taguchi assisted in perioperative care. Mariko Tanaka performed the pathological diagnosis and interpretation.

## Funding

No funding was received for this manuscript.

## Data Availability

The clinical data used to support the findings of this case report are included within the article.
